# Transcript‐Specific DNA Methylation Alterations of the *RASSF1* Locus in Cancer Cells

**DOI:** 10.1002/gcc.70125

**Published:** 2026-04-20

**Authors:** Litzy Gisella Bermudez, Camila Bernal Forigua, Carmen María Ayala‐Roldán, Jesús Manuel Romero, Rafael R. Ariza, Teresa Morales‐Ruiz, Teresa Roldán‐Arjona, Alejandra Cañas, Adriana Rojas

**Affiliations:** ^1^ Faculty of Medicine, Institute of Human Genetics Pontificia Universidad Javeriana Bogotá Bogotá Colombia; ^2^ Department of Genetics University of Córdoba Córdoba Spain; ^3^ Maimónides Biomedical Research Institute of Córdoba (IMIBIC) Córdoba Spain; ^4^ Reina Sofía University Hospital Córdoba Spain; ^5^ Faculty of Medicine, Department of Internal Medicine Pontificia Universidad Javeriana Bogotá Colombia

**Keywords:** DNA methylation, epigenetics, gene expression regulation, neoplasms, *RASSF1*

## Abstract

**Background:**

The locus encoding Ras association domain family member 1 (*RASSF1*) encodes multiple transcripts with opposing roles in cancer, such as *RASSF1A* (tumor suppressor), *RASSF1C* (oncogene), and the lncRNA *RASSF1‐AS1* (function undefined). Although DNA methylation–mediated repression of *RASSF1A* expression has been extensively studied in different cancer types, the epigenetic regulation of *RASSF1C* and *RASSF1‐AS1* is unclear. We profiled gene expression and promoter DNA methylation of *RASSF1A*, *RASSF1C*, and *RASSF1*‐*AS1* across 11 tumor cell lines, quantified *RASSF1A* methylation in lung cancer tissues and plasma by quantitative methylation‐specific PCR (qMSP), and integrated single‐CpG methylation (pyrosequencing) with *in silico* transcription factor binding site (TFBS) prediction.

**Results:**

We found that *RASSF1A* promoter hypermethylation was strongly and inversely associated with its mRNA levels. In contrast, *RASSF1C* promoter methylation did not correlate with expression. *RASSF1‐AS1* showed low promoter methylation accompanied by high expression. In clinical samples, *RASSF1A* methylation was detected in tissue biopsy (54% of cases) and plasma (42% of cases) from lung cancer patients, whereas no methylation was detected in 85% of control individuals, regardless of their smoking history. Finally, the analysis of DNA methylation at specific CpG sites combined with the prediction of TFBS in the evaluated promoter regions allowed the identification of binding domains overlapping differentially methylated regions. Notably, the *RASSF1C* promoter region exhibited a higher TFBS frequency containing CpG sites with low methylation levels, as determined by pyrosequencing.

**Conclusions:**

These findings highlight isoform‐specific epigenetic regulation at the *RASSF1* locus and suggest that *RASSF1A* methylation may represent a promising minimally invasive marker in lung cancer.

**Abbreviations**
5‐AZA: 5‐aza‐2′‐deoxycytidine
5mC: 5methylcytosine
CGIs: CpG islands
CRD: N‐terminal cysteine‐rich domain
CTCF: CCCTC‐binding factor
DNMTs: DNA methyltransferases
FFPE: formalin‐fixed, paraffin‐embedded
FIMO: Find Individual Motif Occurrences
LncRNA: Long noncoding RNA
PRC2: Polycomb repressive complex 2
qMSP: Quantitative methylation‐specific PCR
*RASSF1*
: Ras association domain family member 1
*RASSF1‐AS1*
: RASSF1 Antisense RNA 1
SAM: S‐adenosyl methionine
TFBS: Transcription factor binding site
TNM: Tumor‐node‐metastasis

## 1 Background

Epigenetic mechanisms orchestrate gene expression without altering the DNA sequence in a tissue‐specific manner and in response to the cellular microenvironment [1]. Non‐mutational epigenetic reprogramming facilitates the acquisition of key cancer hallmarks, including sustained proliferation signaling, evasion of apoptosis, invasion, and metastasis [2]. A major epigenetic mechanism is DNA methylation, a covalent modification catalyzed by DNA methyltransferases (DNMTs) that transfer a methyl group from S‐adenosyl methionine (SAM) to the fifth carbon of cytosine residues followed by a guanine (CpG sites) to form 5‐methylcytosine (5mC) [3]. About 2% of CpG sites are clustered into regions called CpG islands (CGIs), DNA sequences longer than 200 nt with a > 60% CpG site content [4]. Notably, around 70% of gene regulatory regions, such as promoters, have at least one CGI DNA methylation within these regions is generally associated with the repression of gene expression by preventing binding of transcriptional activation factors and recruiting repressor proteins with specific domains that recognize 5mC [5].

Deregulation of DNA methylation patterns in cancer occurs at both global and gene‐specific levels. Global hypomethylation has been observed in repetitive genome elements, promoting their retro‐transposable activity and contributing to genomic instability [6]. At the gene level, abnormal patterns of DNA methylation include hypomethylation of oncogenes and hypermethylation of tumor suppressor genes [6]. Specifically, abnormal hypermethylation in promoter regions of genes such as *CDKN2A, MGMT*, and *RASSF1A* is associated with the promotion of apoptosis evasion and sustained cell proliferation in tumor cells [7, 8]. Many of these epigenetic alterations occur in the early stages of tumorigenesis, and given their stability, DNA methylation signatures have become valuable biomarkers for diagnostic and predictive use in different cancer models like lung and breast cancer [9].

DNA methylation in *RASSF1A* has been studied and reported as a biomarker in cancer [10]. However, this gene is part of a complex locus at 3p21.31 (*RASSF1*) that includes several regulatory regions and expresses multiple transcripts (*RASSF1A*–*RASSF1H*) [10]. *RASSF1A* and *RASSF1C* are transcribed from independent promoter regions containing independent CGIs, whereas *RASSF1B‐, ‐D, ‐E, ‐F, ‐G*, and *‐H* arise from alternative splicing events of the *RASSF1A* primary transcript. Additionally, *RASSF1‐AS1*, an antisense long noncoding RNA (lncRNA), is transcribed from the complementary strand and shares its CGI with *RASSF1C* [11].

*RASSF1A and RASSF1C* encode two protein isoforms that, despite being structurally close, are two antagonists during carcinogenesis [12]. *RASSF1A* is a tumor suppressor that acts as a scaffolding protein through its N‐terminal cysteine‐rich domain (CRD), allowing the recruitment of specific proteins and the regulation of downstream signaling pathways. It controls cell cycle progression through its association with the microtubule network and the inhibition of cyclins A and B [9], and in addition it promotes apoptosis by activating the death receptor complex (TNF‐R1/MOAP‐1 or TRAIL‐R1/MOAP‐1) [10]. In contrast, *RASSF1C* does not have a CRD domain and actively participates in promoting tumor cell proliferation, migration, and survival in breast and lung cancer models. Although the mechanisms underlying its oncogenic activity remain unclear, it has been shown that *RASSF1C* regulates microRNA expression in cancer [13].

Although hypermethylation of the *RASSF1A* promoter region has been extensively described in cancer [14, 15], the impact of aberrant DNA methylation on the coordinated expression of the *RASSF1A*, *RASSF1C* isoforms and the *RASSF1‐AS1* antisense transcript remains underexplored, despite its potential functional relevance. In this study, we aimed to characterize the methylation profiles of *RASSF1A*, *RASSF1C*, and *RASSF1A‐AS1* regulatory regions, and evaluate their association with their gene expression profiles in tumor cell lines of different origins. Our findings established that promoter DNA methylation is a key epigenetic regulator of transcription for both *RASSF1A* and the antisense lncRNA *RASSF1‐AS1*. To further investigate these observations and explore their detectability in clinical settings, we analyzed *RASSF1A* promoter methylation in tumor tissue and liquid biopsy samples from lung cancer patients [16].

## 2 Methods

### 2.1 Cell Culture

The following human tumor cell lines were used: A549 (lung cancer, ATCC CCL‐185) and H23 (lung cancer, ATCC CRL‐5800); A172 (glioblastoma, ATCC CRL‐1620), LN229 (glioblastoma, ATCC CRL‐2611), T98G (glioblastoma, ATCC CRL‐1690), and U373 (glioblastoma, also known as U‐373 MG, Sigma‐Aldrich 08061901); DLD‐1 (colorectal cancer, ATCC CCL‐221) and HCT116 (colorectal cancer, ATCC CCL‐247); MCF7 (breast cancer, ATCC HTB‐22) and HeLa (cervical cancer, ATCC CCL‐2). Two non‐tumorigenic human cell lines were also included: HEK‐293 (ATCC CRL‐1573), derived from human embryonic kidney cells and widely used for gene expression studies, and HDFa (Cat. No. C‐013‐5C, Invitrogen), adult human dermal fibroblasts. All cell lines were cultured in appropriate media supplemented with 10% fetal bovine serum and 1% penicillin–streptomycin, following the specific recommendations provided by the corresponding suppliers. All cell cultures were incubated at 37°C in 5% CO_2_ with a relative humidity of 95%. Cells were harvested upon reaching 80%–90% confluence.

### 2.2 Tissue and Liquid Biopsy Samples From Lung Cancer Cases

A total of 13 formalin‐fixed, paraffin‐embedded (FFPE) lung tumor biopsies collected at Hospital Universitario San Ignacio between 2016 and 2023 were included in the study. Histopathological diagnosis of lung cancer was confirmed, and tumor‐node‐metastasis (TNM) staging was assigned according to the guidelines of the American Joint Committee on Cancer and the International Union Against Cancer (Table 1). Liquid biopsy samples were collected at Hospital Universitario Reina Sofía between 2012 and 2015 from a cohort of 12 lung cancer cases and 26 individuals without evidence of lung pathology (17 smokers, 9 no smokers) (Table 2). Peripheral blood was drawn from each participant, and the plasma fraction was separated for subsequent experimental analysis. The study was approved by the Ethics Committee of the Faculty of Medicine at Pontificia Universidad Javeriana (FM‐CIE‐0821‐24) and Ethics Committee of the Reina Sofia University Hospital (26/07/2012). All procedures were conducted following written informed consent obtained from each participant.

**TABLE 1:** Clinical characteristics of a cohort of lung cancer cases with paraffin‐embedded tissue biopsy.

Group	Lung cancer cases
Total individuals	13 (percentage)
Female	9 (69%)
Male	4 (31%)
Median age (years range)	63 (29–85)
Histological tumor type	
Adenocarcinoma	8 (61.5%)
Squamous cell carcinoma	3 (23.1%)
Carcinoid tumor	2 (15.4%)
Large cell carcinoma	0 (0%)
Small cell carcinoma	0 (0%)
No information	0 (0%)
TNM stage	
I	6 (46.2%)
II	2 (15.4%)
III	1 (7.7%)
IV	4 (30.8%)
No information	0 (0%)
Exposure history	
Smokers	6 (46%)
None	0 (54%)

**TABLE 2:** Clinical characteristics of cohort of individuals with liquid biopsy.

Group	Lung cancer cases	Control individuals
Total individuals	12 (percentage)	26 (percentage)
Female	0 (0%)	4 (15%)
Male	12 (100%)	22 (85%)
Median age (years range)	61 (50–70)	58 (43–81)
Histological tumor type		
Adenocarcinoma	2 (16.67%)	—
Squamous cell carcinoma	2 (16.67%)	—
Carcinoid tumor	4 (33.33%)	—
Large cell carcinoma	1 (8.33%)	—
Small cell carcinoma	1 (8.33%)	—
No information	2 (16.67%)	—
TNM stage		
I	1 (8.33%)	—
II	0 (0%)	—
III	3 (25%)	—
IV	6 (50%)	—
No information	2 (16.67%)	—
Exposure history		
Smokers	12 (100%)	17 (65%)
None	0 (0%)	9 (35%)

### 2.3 Reverse Transcription and Quantitative Real‐Time PCR (qPCR)

Total RNA was extracted from cells using TRIzol reagent (cat. no. 15596026, Ambion, Life Technologies), and from tumor tissue samples, extraction was performed using the Quick‐DNA/RNA FFPE Kit (Cat. no. R1009, Zymo Research) according to the manufacturer's instructions. Subsequently, 2 μg of total RNA, quantified using a NanoDrop 2000c spectrophotometer (Thermo Fisher Scientific), was used for cDNA synthesis with the iScript cDNA Synthesis Kit (Cat. no. 1708890, Bio‐Rad), following the manufacturer's protocol. Gene expression levels of *RASSF1A*, *RASSF1C*, and *RASSF1‐AS1* were assessed by quantitative PCR using the NZYSupreme qPCR Green Master Mix (2×) (Cat. no. MB22402, NZY Tech) and the CFX Connect Real‐Time PCR Detection System (Bio‐Rad). The thermal cycling conditions were: initial denaturation at 95°C for 10 min, followed by 45 cycles of denaturation at 95°C for 10 s, primer annealing for 15 s at gene‐specific temperatures (Table S1), and extension at 72°C for 20 s. Expression of protein‐coding genes was normalized to *GAPDH* (cell lines) and 18S rRNA (formalin‐fixed, paraffin‐embedded tissue samples‐FFPE), while expression of the long non‐coding RNA *RASSF1‐AS1* was normalized to *U6*, a small nuclear RNA involved in spliceosomal function. These normalization strategies were applied independently according to transcript type and origin and preservation method of sample and were not combined across datasets. Δ*Ct* values were calculated independently within each dataset using the corresponding endogenous control.

### 2.4 Methylation Analysis by Pyrosequencing and Quantitative Methylation‐Specific PCR (qMSP)

Genomic DNA was extracted from cells and plasma using the QIAamp DNA Blood and Other Fluids kit (Cat. No. 51194, QIAGEN), and from FFPE tissue samples using the Quick‐DNA/RNA FFPE kit (Cat. No. D3067, Zymo Research). Approximately 500 ng of DNA were bisulfite‐converted using the EZ DNA Methylation‐Gold (Cat. No. D5005, Zymo Research). Bisulfite‐treated DNA was subsequently amplified by PCR using the MyFi DNA Polymerase (Cat. No. BIO‐21117, BIOLINE) and biotinylated primer sets specific to the promoter regions of *RASSF1A*, *RASSF1C*, and *RASSF1‐AS1* (Table S1). Pyrosequencing was carried out with the PyroMark Q24 system using the PyroMark Q24 Advanced CpG Reagents (Cat. No. 970922, QIAGEN), and methylation levels at individual CpG sites were quantified with the PyroMark Q24 software. Methylation‐specific PCR was performed using primers targeting the methylated sequence of the *RASSF1A* promoter and primers targeting the *β‐ACTIN* sequence as the input DNA control (Table S1).

### 2.5 5‐Aza‐2′‐Deoxycytidine (5‐Aza‐dC) Treatment

To evaluate the effect of DNA demethylation on gene expression, A549 and H23 lung cancer cell lines were treated with 5‐aza‐2′‐deoxycytidine (5‐aza‐dC; Sigma‐Aldrich, Cat. No. A3656) at a final concentration of 5 μM. Cells were initially seeded at a density of 1 × 10^5^ cells per well in 6‐well plates (Day 0). From day one, cells were treated daily for five (A549) or three (H23) consecutive days with freshly prepared culture medium containing vehicle (DMSO) or 5‐aza‐dC. At the end of treatment, cells were collected for DNA and RNA extraction to assess the impact of demethylation on *RASSF1A* expression.

### 2.6 Identification of Critical CpG Sites and Transcription Factor Binding Site Prediction

To identify CpG sites with potential regulatory relevance, a relative methylation index was calculated for each CpG site by dividing its individual methylation value by the mean promoter methylation level within the same cell line. This analysis was performed separately for *RASSF1A* (9 CpG sites), *RASSF1C* (20 CpG sites), and *RASSF1‐AS1* (22 CpG sites). Subsequently, the bisulfite‐pyrosequenced genomic regions (*RASSF1A*: 135 bp, *RASSF1C*: 301 bp, and *RASSF1‐AS1*: 299 bp) were analyzed using the FIMO (Find Individual Motif Occurrences) tool within the MEME Suite, querying transcription factor binding motifs from the JASPAR 2024 CORE vertebrate database [17]. Predicted transcription factor binding sites (TFBS) were mapped across the same genomic intervals analyzed by pyrosequencing. Only statistically significant TFBS overlapping at least one CpG dinucleotide were retained for downstream analysis. For each CpG site evaluated by pyrosequencing, the frequency of predicted TFBS directly containing that CpG within their binding motif was calculated and used to identify CpG positions with potential regulatory relevance.

### 2.7 Statistical Analysis

Non‐parametric statistical tests were applied to evaluate differences between the analyzed groups: the Mann–Whitney test for comparisons between two independent groups, the Kruskal‐Wallis test for comparisons among three or more independent groups, and Spearman's correlation to assess the relationship between mRNA expression levels (Δ*Ct* values) and promoter methylation of *RASSF1A*, *RASSF1C*, and *RASSF1‐AS1*. In addition, heatmap visualization and clustering analyses were performed in R (version 4.4.3). Net Δ*Ct* values obtained from the qPCR experiments were used as input without further transformation or normalization between genes or cell lines. Unsupervised hierarchical clustering was applied to classify cell lines according to their relative expression profiles of *RASSF1A*, *RASSF1C*, and *RASSF1‐AS1*. Clustering was performed using Euclidean distance as the dissimilarity measure and the complete linkage method. Heatmaps were generated using the ComplexHeatmap package [18], allowing visualization of expression patterns and grouping of cell lines with similar transcriptional profiles. All statistical analyses were conducted using GraphPad Prism version 8 (GraphPad Software, California, USA) and R version 4.4.3. Graphs show the median with 95% confidence intervals. All experiments involving cell lines were performed with three independent biological replicates (*n* = 3).

## 3 Results

**FIGURE 1:**
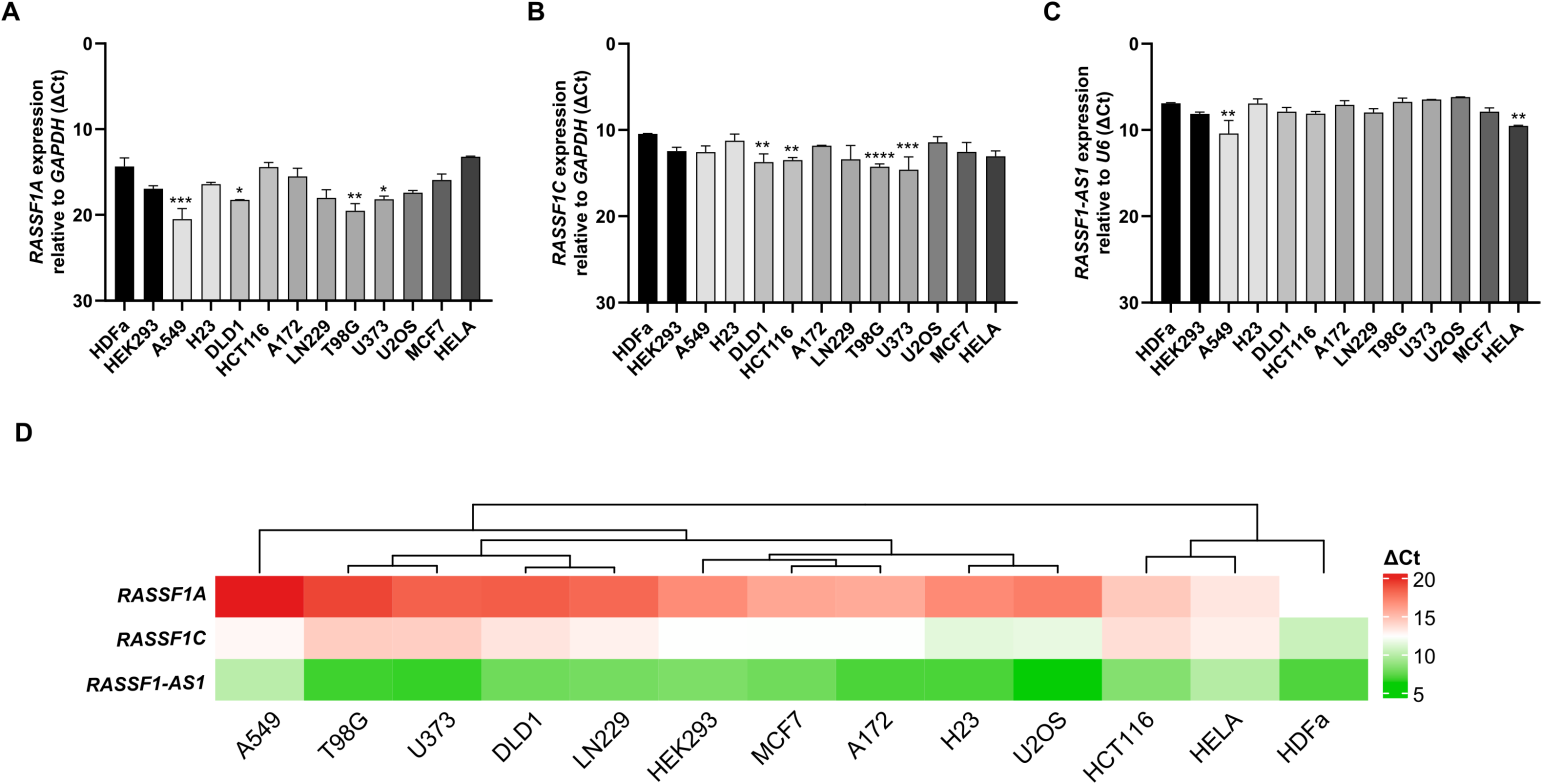
Expression profiles of *RASSF1A*, *RASSF1C*, and *RASSF1‐AS1* in tumor cell lines. (A–C) mRNA expression levels of *RASSF1A*, *RASSF1C*, and *RASSF1‐AS1*. All expression values are expressed as Δ*Ct*, where lower Δ*Ct* corresponds to higher gene expression. Statistically significant differences with non‐tumor HDFa cells are indicated by asterisks (*p*‐value were < 0.0332 (*), < 0.0021 (**), < 0.0002 (***), and < 0.0001 (****)). (D) Unsupervised hierarchical clustering heatmap of cell lines based on the relative expression levels of *RASSF1A*, *RASSF1C*, and *RASSF1‐AS1*. Each column represents a cell line, and each row corresponds to one of the three transcripts. Color intensity reflects normalized Δ*Ct* values: Red indicates high Δ*Ct* (20), while green indicates low Δ*Ct* (5). No additional normalization or transformation was applied prior to heatmap construction.

To profile *RASSF1A*, *RASSF1C*, and *RASSF1*‐AS1 transcription across the tumor cell‐line panel, we performed qPCR and reported Δ*Ct* values relative to the reference gene (lower Δ*Ct* = higher expression). Using the Δ*Ct* distribution, samples were stratified into tertiles, and cutoffs were defined at the 33rd (9.083) and 66th (15.308) percentiles to classify three expression levels: high, Δ*Ct* ≤ 9.083; intermediate, 9.083 < Δ*Ct* < 15.308; and low, Δ*Ct* ≥ 15.308. By these criteria, expression levels were predominantly low for *RASSF1A* (Figure 1A), intermediate to high for *RASSF1C* (Figure 1B), and high for *RASSF1‐AS1* (Figure 1C). Relative to the non‐tumor HDFa cells, *RASSF1A* mRNA was significantly reduced in A549 (lung cancer), DLD1 (colon cancer), and T98G and U373 (glioblastoma) cell lines (Figure 1A). *RASSF1C* expression was also significantly lower in the colon cancer lines DLD1 and HCT116 and in T98G and U373 cells (Figure 1B). By contrast, *RASSF1‐AS1* expression level was broadly high across the panel, except in A549 (lung cancer) and HeLa (cervical cancer) cell lines, which showed significantly decreased levels (Figure 1C). To explore possible expression patterns among transcripts derived from the *RASSF1* locus, unsupervised hierarchical clustering of Δ*Ct* values was performed and visualized using a heatmap (Figure 1D). The analysis identified five main clusters of cell lines based on their transcriptional profiles. Cluster 1 (A549, T98G, U373, DLD1, and LN229) was characterized by the low expression of *RASSF1A* and *RASSF1C*, and high expression of *RASSF1‐AS1*. Cluster 2 (HEK‐293, MCF7, and A172) showed low *RASSF1A*, intermediate *RASSF1C*, and high *RASSF1‐AS1* expression. Cluster 3 (H23 and U2OS) showed low *RASSF1A*, high *RASSF1C*, and high *RASSF1‐AS1* expression. Cluster 4 (HCT116 and HeLa) displayed intermediate *RASSF1A*, low *RASSF1C*, and high *RASSF1‐AS1* levels. Finally, Cluster 5 (HDFa) was the only cell line with high expression of *RASSF1A*, *RASSF1‐AS1* and *RASSF1C*. These divergent expression profiles indicate locus‐wide, cell line–specific regulation of *RASSF1* transcripts, consistent with distinct epigenetic configurations in each cellular context.

**FIGURE 2:**
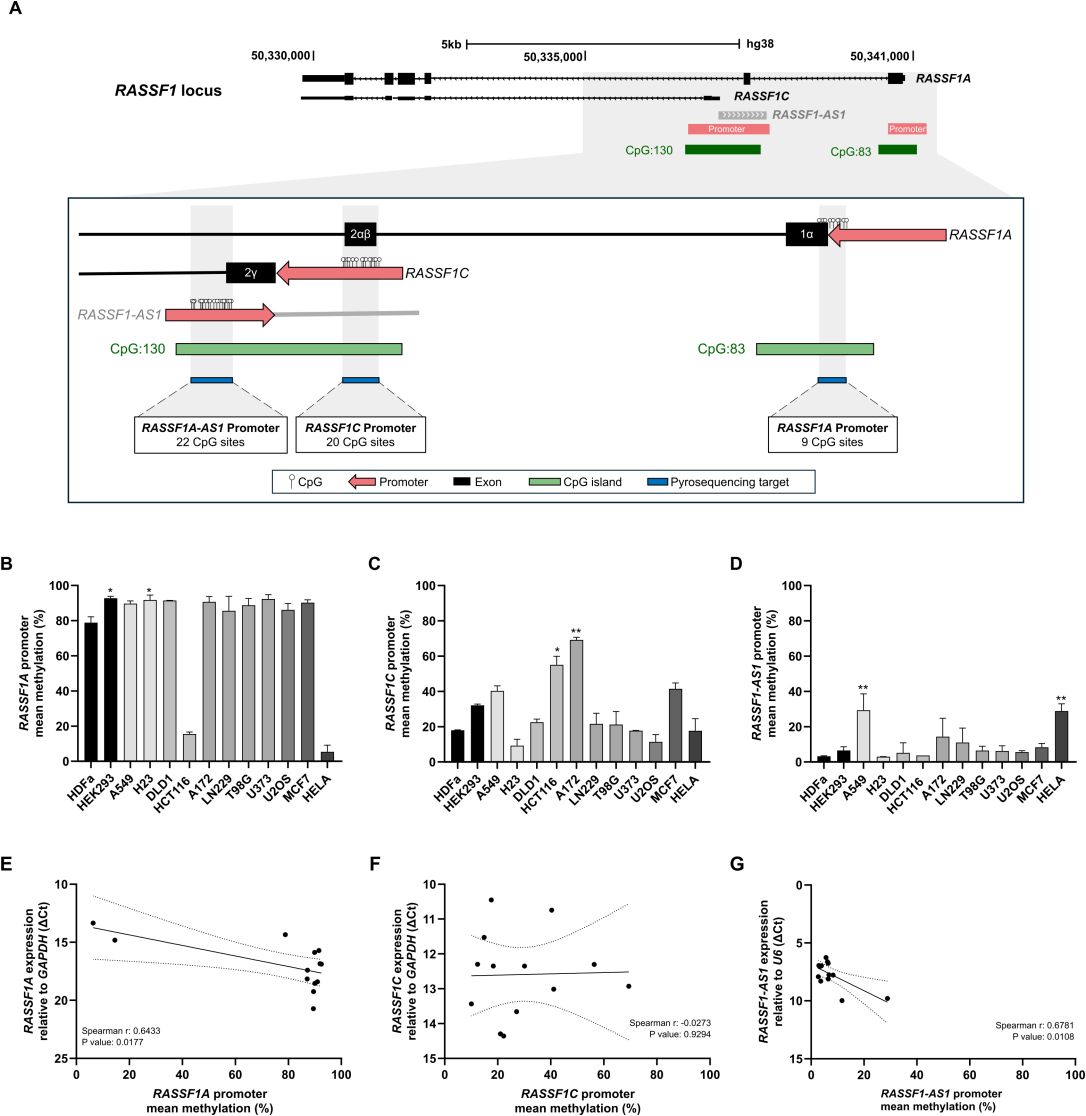
DNA methylation analysis of the regulatory regions of *RASSF1A*, *RASSF1C*, and *RASSF1‐AS1* in tumor cell lines. (A) Schematic representation of the *RASSF1* locus, including gene structure, regulatory regions, CpG islands and pyrosequencing target sequences. Illustration adapted from https://genome.ucsc.edu/. (B–D) Mean methylation percentage by DNA pyrosequencing assays of *RASSF1A* (B), *RASSF1C* (C) and *RASSF1‐AS1* (D) promoter regions. Statistically significant differences with non‐tumor HDFa cells are indicated by asterisks (*p*‐value values were < 0.0332 (*), < 0.0021 (**), < 0.0002). All experiments were performed using three independent biological replicates (*n* = 3) (E–G) Correlation between promoter methylation and mRNA levels of *RASSF1A* (E), *RASSF1C* (F) and *RASSF1‐AS1* (G). Spearman correlation coefficients (*r*) and corresponding *p*‐values are indicated in each panel.

To investigate regulatory mechanisms governing transcription at the *RASSF1* locus, we interrogated genome‐browser tracks and cataloged predicted regulatory elements. This analysis identified three putative promoter regions, each associated with *RASSF1A*, *RASSF1C* and *RASSF1‐AS1* (Figure 2A). In addition, there are two CGI: one located in the promoter region of *RASSF1A*, and another comprising the promoter regions corresponding to *RASSF1C* and *RASSF1‐AS1* (Figure 2A), suggesting possible DNA methylation‐mediated epigenetic regulation. Consequently, DNA pyrosequencing assays were designed (Figure 2A) and applied to the cell lines panel to analyze the methylation profiles of each regulatory region concomitant to the CpG islands.

Methylation levels at the *RASSF1A* promoter region were above 85% in most tumor cell lines, except for HCT116 and HeLa (Figure 2B). By contrast, methylation at the *RASSF1C* promoter was more variable, remaining below 50% in most cell lines except for HCT116 and A172 (Figure 2C). The *RASSF1‐AS1* promoter region exhibited consistently low methylation, with average levels below 20%, except in A549 and HeLa (Figure 2D). To test whether promoter methylation was associated with transcript abundance across cell lines, we computed Spearman rank correlations between promoter methylation and gene expression for each transcript. A significant negative correlation was observed between methylation and gene expression for both *RASSF1A* and *RASSF1‐AS1* (Spearman *r*: 0.643 and 0.678, respectively, *p* value < 0.05) (Figure 2E,G), supporting DNA methylation–mediated epigenetic control. Notably, *RASSF1A* showed high methylation levels associated with transcriptional silencing, while *RASSF1‐AS1* exhibited low methylation levels associated with high expression. In contrast, no correlation was found between *RASSF1C* expression and the methylation status of its promoter region (Figure 2F). Together, these data indicate that *RASSF1A* and *RASSF1‐AS1* are epigenetically regulated by promoter methylation, albeit with distinct methylation–expression relationships. By contrast, the lack of a methylation–expression correlation for *RASSF1C* suggests that its transcription is controlled by mechanisms other than DNA methylation.

**FIGURE 3:**
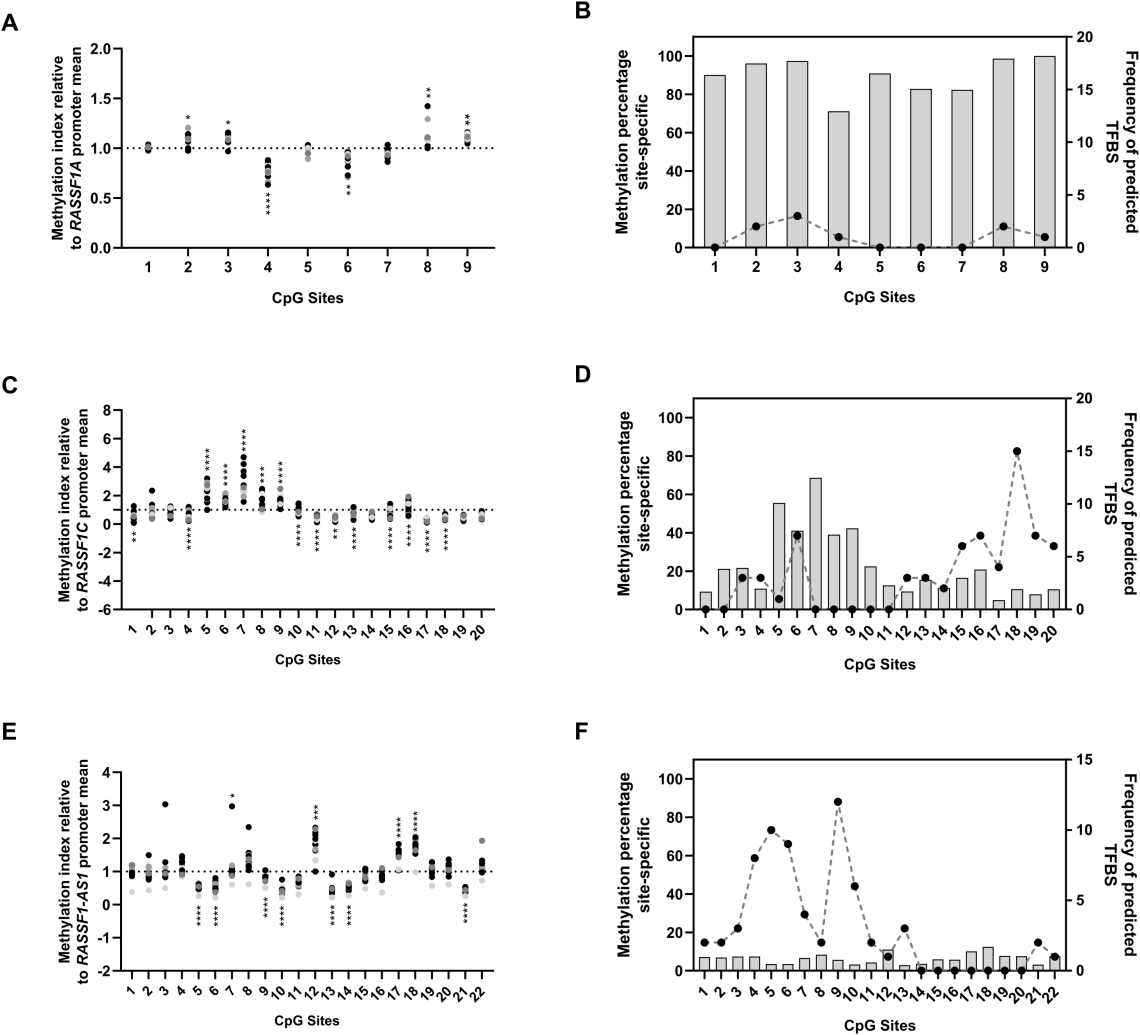
Identification of critical CpG sites for methylation and analysis of transcription factor binding motifs in the promoter regions of *RASSF1A*, *RASSF1C*, and *RASSF1‐AS1*. Relative methylation index of individual CpG sites, calculated as the ratio of the percentage of methylation at each site to the average promoter methylation within the same cell line, for *RASSF1A* (A), *RASSF1C* (C), and *RASSF1‐AS1* (E). CpG sites with values greater than 1 were considered to exhibit relatively higher methylation compared with the promoter mean. Statistical significance *p*‐value values were < 0.0332 (*), < 0.0021 (**), < 0.0002 (***), and < 0.0001 (****). *In silico* prediction of the frequency of transcription factor binding motifs (dotted line) overlapping with the percentage of methylation at individual CpG sites (gray bars) in the promoter regions of *RASSF1A* (B), *RASSF1C* (D), and *RASSF1‐AS1* (F).

To assess whether there are critical CpG sites with differential methylation relative to the average levels across the analyzed promoter regions, and consequently with a potential impact on gene expression, a relative methylation index was calculated for each CpG site (Figure 3). Additionally, the genomic distribution of the analyzed CpG sites within the *RASSF1* locus for the *RASSF1A* and *RASSF1C* isoforms, as well as the lncRNA *RASSF1‐AS1*, is shown (Figure S1). In the *RASSF1A* promoter region, CpG sites 2, 3, 8, and 9 exhibited methylation levels consistently higher than the mean promoter methylation in the corresponding cell lines across the panel, suggesting localized hypermethylation at these positions. In contrast, CpG sites 4 and 6 generally showed lower methylation levels relative to the regional average within the same cell lines (Figure 3A). Interestingly, the *RASSF1C* promoter region showed bimodal methylation pattern at the CpG site‐specific level: CpG sites 5–9 evidenced higher methylation levels of up to fourfold over the average, whereas sites such as 1, 4 and 10 to 18 generally exhibited low methylation levels in the cell line panel (Figure 3C). In the *RASSF1‐AS1* promoter region most CpG sites did not differ from the average methylation, but CpG sites 5, 6, 9, 10, 13, 14 and 21 showed a trend of lower methylation and CpG sites 12, 17 and 18 showed a trend of higher methylation levels, being the only CpG sites that varied in cell lines (Figure 3E).

We then scanned the promoter regions with FIMO (MEME Suite) to identify TFBS that overlap the analyzed CpGs (Table S2). A total of 5 TFBS containing one or more CpG sites evaluated in the *RASSF1A* promoter region were identified (Figure 3B). Interestingly, most of these TFBS co‐localize with critical highly methylated CpG sites in the promoter region. On the other hand, 30 out of 39 TFBS identified in the analyzed promoter region of *RASSF1C* contained one of the critical sites with lower methylation (Figure 3D). Meanwhile, 28 TFBS were identified in the analyzed regulatory region of *RASSF1*‐*AS1*. Most of them mainly contained CpG sites with levels equal to or lower than the average hypomethylation of the region (Figure 3F).

**FIGURE 4:**
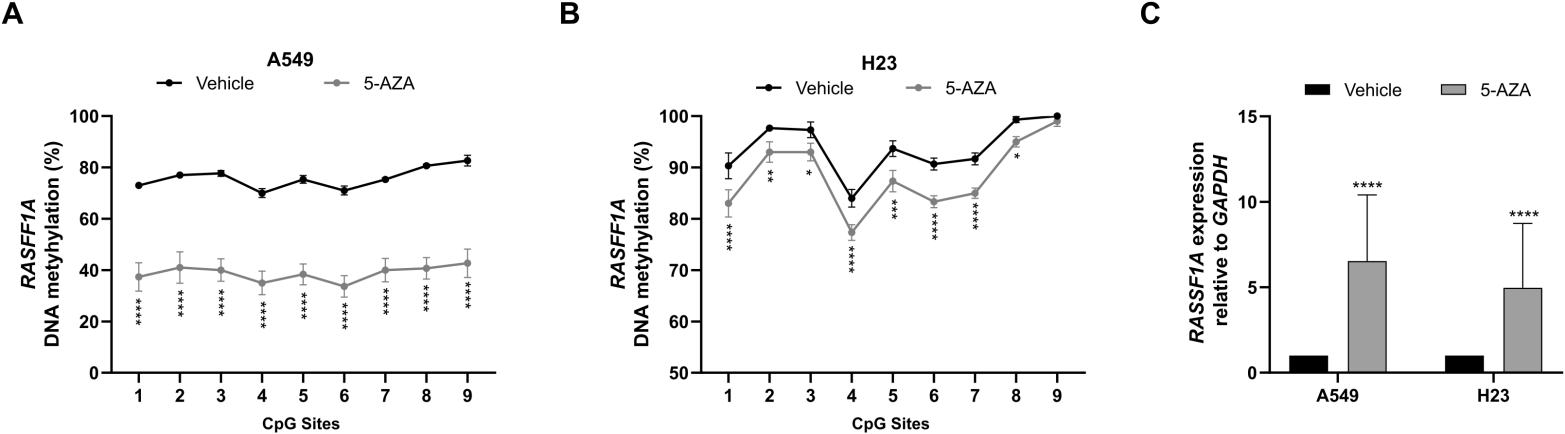
Effect of DNA hypomethylation induced by 5‐Aza‐2′‐deoxycytidine on *RASSF1A* expression in lung cancer cell lines. Specific CpG site methylation within the *RASSF1A* promoter region in A549 (A) and H23 (B) cells treated with 5‐Aza‐dC (5‐AZA), compared to vehicle‐treated control cells (Vehicle). (C) Relative expression levels of *RASSF1A* in A549 and H23 cells treated with 5‐Aza‐dC compared to vehicle‐treated control cells. Statistical significance *p*‐value values were < 0.0332 (*), < 0.0021 (**), < 0.0002 (***), and < 0.0001 (****). Data are presented as median values from three independent biological replicates (*n* = 3).

Given the potential clinical relevance of *RASSF1A* promoter hypermethylation in lung cancer [15], we performed 5‐aza‐2′‐deoxycytidine (5‐aza‐dC) demethylation assays in lung cancer cell lines to assess the methylation dependence of gene expression (Figure 4). Incorporation of 5‐aza‐dC into DNA during replication and its subsequent irreversible binding to DNMTs leads to their depletion and results in global DNA hypomethylation. We found that treatment with 5‐aza‐dC in A549 and H23 cell lines significantly reduced methylation levels at CpG sites within the *RASSF1A* promoter region (Figure 4A,B). Notably, these lower methylation levels were associated with a significant increase in *RASSF1A* mRNA expression in both treated cell lines compared to vehicle‐treated control cells (Figure 4C). These results support a functional link between promoter methylation and *RASSF1A* transcriptional repression.

**FIGURE 5:**
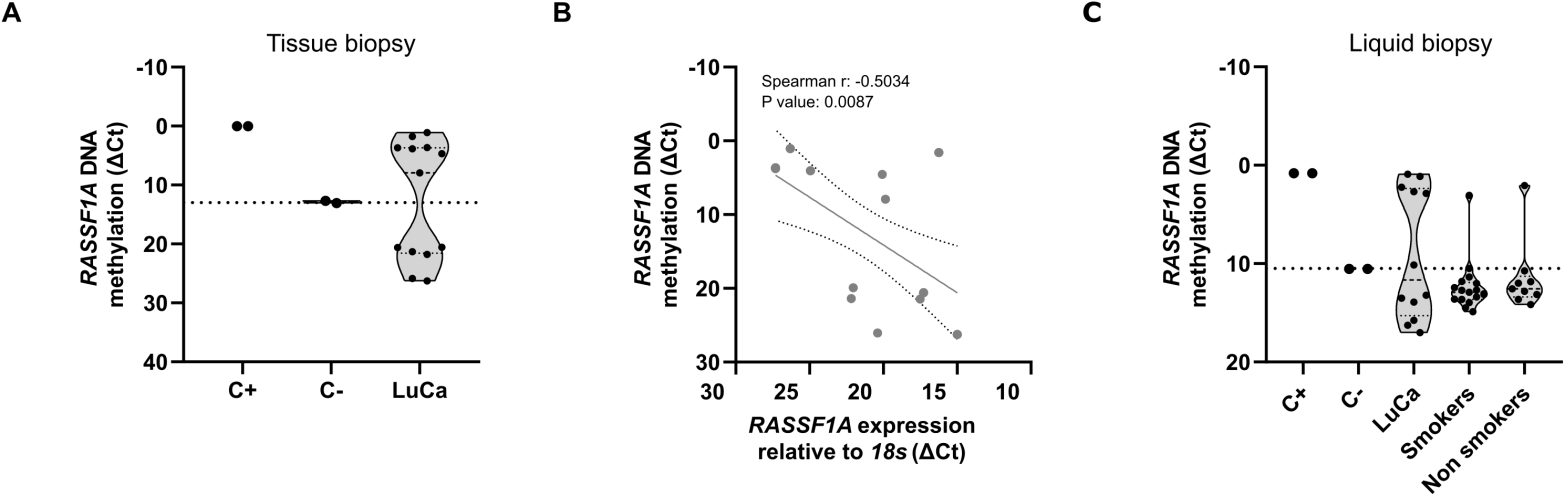
Analysis of *RASSF1A* promoter methylation in samples derived from lung cancer patients. (A) Methylation levels of the *RASSF1A* promoter in formalin‐fixed, paraffin‐embedded tumor tissue samples from lung cancer patients. Methylation levels were assessed by qMSP and expressed as Δ*Ct* values, where lower Δ*Ct* indicates higher methylation. Methylated (C+) and unmethylated (C−) DNA controls were included. Samples with Δ*Ct* values lower than those observed in the C− control were considered within the background range and therefore negative for detectable methylation. (B) Correlation between *RASSF1A* promoter methylation and gene expression levels in tumor tissue samples. Spearman correlation coefficient (*r*) and corresponding *p*‐value are indicated in the panel. (C) Methylation levels of the *RASSF1A* promoter in liquid biopsy samples from lung cancer patients and control individuals (smokers and non‐smokers).

Given the consistent repression of *RASSF1A* by promoter methylation observed in lung cancer cell lines, we examined this mark in patient samples and evaluated its detectability in both tumor tissues and liquid biopsies. For this objective, methylation of the *RASSF1A* promoter was analyzed by qMSP in tumor tissue and plasma samples from patients diagnosed with lung cancer. Methylation of the *RASSF1A* promoter was detected in 54% (7/13) of tumor tissue samples (Figure 5A). Subsequently, RNA was extracted from the same tissue section, and the mRNA expression levels of *RASSF1A* were evaluated. Interestingly, these expression levels showed a significant negative correlation with the detected methylation levels (Figure 5B). *RASSF1A* methylation was also detected in 42% (5/12) of plasma samples from lung cancer patients (Figure 5C). In contrast, more than 85% of control individuals, regardless of tobacco smoke exposure, showed no detectable *RASSF1A* methylation. Because methylation levels in qMSP are usually calculated using relative quantification approaches (Δ*Ct* normalization to a reference gene), small variations in amplification efficiency or normalization may occasionally produce values in experimental samples that appear slightly lower than the signal detected in the unmethylated control. Importantly, such values generally fall within the background detection range of the assay and therefore do not represent biologically meaningful methylation but rather technical variation around the baseline signal. Similar observations have been reported in previous qMSP studies and are considered a common feature of highly sensitive methylation‐specific PCR–based methods [19, 20, 21]. To complement the methylation patterns observed in the experimental cohorts, DNA methylation levels at CpG sites within the *RASSF1A* promoter region were assessed using publicly available datasets from The Cancer Genome Atlas (TCGA) through the SMART App platform [22] in lung adenocarcinoma (LUAD, *n* = 486) and lung squamous cell carcinoma (LUSC, *n* = 412) cohorts. These analyses revealed significant changes in *RASSF1A* promoter methylation in lung cancer cases, supporting the patterns observed in the experimental samples (Figure S2). Furthermore, to explore whether these methylation patterns were present in early stages of the disease, the same CpG sites were evaluated across pathological stages (I–IV). Overall, aberrant *RASSF1A* methylation levels were observed regardless of stage at diagnosis, suggesting that these epigenetic alterations occur throughout disease progression and are not limited to advanced‐stage tumors (Figure S3). Taken together, these data show that aberrant *RASSF1A* promoter methylation is detectable in samples from lung cancer patients.

## 4 Discussion

This study identified differential DNA methylation patterns of *RASSF1A*, *RASSF1C*, and *RASSF1‐AS1* regulatory regions, suggesting isoform‐ and lncRNA‐specific epigenetic regulation of the *RASSF1* locus in a tumor context. We found that the *RASSF1A* promoter was hypermethylated, with concordant transcriptional silencing, in most cell lines, and this effect was reversed by 5‐aza‐2′‐deoxycytidine, which reactivated *RASSF1A* expression in A549 and H23 lung cancer cell lines. Epigenetic silencing of *RASSF1A* has been widely reported in cancer models such as lung, breast, colon, and glioblastoma [23, 24, 25, 26, 27]. It has been demonstrated that *RASSF1A* promoter hypermethylation in cancer is promoted by the homeobox protein HOXB3 by increasing the expression of DNA methyltransferases DNMT1 and DNMT3B, which are subsequently recruited to the *RASSF1A* promoter region through interactions with the Polycomb repressive complex 2 (PRC2), the MUC1‐C‐ZEB1 complex, and the transcription factor MYC [28, 29, 30]. Furthermore, it has been reported that DNA methylation in the *RASSF1A* promoter region leads to gene silencing by decreasing the binding affinity of transcription factors such as 11‐zinc finger protein [31].

This study identified critical CpG sites with above‐average methylation levels in the analyzed region located between the promoter and the first exon of *RASSF1A*, whose position overlaps with predicted binding domains for transcription factors such as ZBED4, KLF15, ZNF213, ZNF449, and THRA. Interestingly, DLD1 and HCT116 colon cancer cell lines exhibited opposite methylation patterns, with less than 20% observed for the latter. These findings differ from those reported by Cheng et al., where *RASSF1A* hypermethylation in HCT116 was associated with gene expression repression, an effect that was reversed by treatment with 5‐aza‐dC [32]. However, the authors did not disclose details about the exact region analyzed. On the other hand, the hypomethylation of *RASSF1A* in the HeLa cell line is consistent with the findings of Rammann et al. [33] indicating that *RASSF1A* promoter is in an active chromatin state characterized by high levels of histone acetylation and the absence of repressive marks such as H3K9me2, which prevents the recruitment of DNA methyltransferases and, consequently, DNA methylation in the HeLa cell line. These findings highlight the critical role of the chromatin context in regulating *RASSF1A* promoter methylation, potentially explaining the distinct DNA methylation patterns observed across different cellular models, such as HeLa and HCT116, for this tumor suppressor gene.

We found variable DNA methylation levels in the *RASSF1C* promoter region across different cell lines. Most cell lines had average DNA methylation levels in the promoter below 50%, which is consistent with reports in the literature on low methylation levels of this isoform in models such as breast cancer, pancreatic cancer, and pituitary adenoma [11, 34, 35]. Interestingly, the average DNA methylation levels of the promoter region did not correlate with gene expression levels. High levels of *RASSF1C* expression have been reported in cancer models such as lung, thyroid, and breast cancer [36, 37].

When evaluating methylation levels at the CpG site‐specific level, heterogeneity was identified in the distribution of DNA methylation marks across the 20 CpG sites evaluated, with significantly lower DNA methylation status in the region near the TSS +1 site (CpG 12–20) of *RASSF1C* (Figure S1). In addition, this region has a high frequency of transcription factors binding domains according to the prediction analysis. Interestingly, a detailed review of the TFBS prediction revealed that CpG sites where DNA methylation levels begin to decrease within the analyzed *RASSF1C* region (e.g., CpG15, Figure 3D) overlap with a predicted CTCF binding site (Table S2). CTCF is a key architectural protein known to mediate insulator‐like functions by establishing chromatin boundaries that restrict regulatory interactions and the propagation of epigenetic marks [38]. During DNA replication, CpG methylation patterns are primarily maintained by DNMT1, which restores parental methylation states on newly synthesized DNA strands and can promote the local propagation of methylation across adjacent CpG sites [39, 40]. In this context, the presence of a predicted CTCF binding site near the observed transition from highly methylated to low‐methylated CpGs suggests that this region may act as a local epigenetic boundary within the *RASSF1* locus. Such boundaries may also interact with other epigenetic features, including histone modifications associated with active chromatin states such as H3K4 methylation, which are known to inhibit DNA methyltransferase recruitment [41]. Together, these mechanisms may contribute to the CpG‐specific methylation variability observed in the *RASSF1C* region.

These findings highlight the importance of evaluating methylation at the site‐specific level to understand its potential impact on gene regulation, since the overall methylation status of the promoter could mask epigenetic alterations located at key sites for transcription factor access and, therefore, for *RASSF1C* transcriptional activation. Consequently, the difficulty in kinetically capturing peaks or troughs of methylation within narrow genomic intervals may reflect the combined effects of replication‐coupled maintenance methylation, insulator‐like chromatin boundaries, and cellular heterogeneity within the analyzed cell populations.

To date, no experimental evidence has shown transcription factor binding at the *RASSF1C* promoter in cancer. However, it has been shown that demethylation induced by 5‐Aza‐2′‐deoxycytidine does not affect the expression of this transcript, highlighting the need to explore additional epigenetic mechanisms involved in the regulation of this oncogene [11].

We observed significant correlations between hypomethylation of the *RASSF1‐AS1* promoter region and its high expression levels across the panel of tumor cell lines analyzed, which included lung, colon, glioblastoma, osteosarcoma, breast, and cervical cancer lines. Although literature reports on the expression and DNA methylation of this transcript remain scarce, our findings are consistent with those described by Calanca et al. and Beckedorff et al. in studies evaluating its expression and methylation status in breast cancer [11, 42]. Furthermore, they observed that demethylation induced by treatment with Aza‐2′‐deoxycytidine did not alter the expression of this transcript [42]. Specific analysis of CpG sites revealed a low level of variability in terms of hypomethylation along the *RASSF1‐AS1* promoter. Furthermore, although 28 predicted transcription factor binding sites were identified in this region, no reports were found on the transcriptional regulation of this lncRNA. In 2019, Heilman and colleagues reported that DNA methylation in enhancers predominated over promoters in the impact on lncRNA gene expression in breast cancer [43]. Meanwhile, covalent modifications of histones and the presence of transcription factor domains have been characterized as the main mechanisms determining transcriptional activity in lncRNA promoters [44, 45].

Our study observed higher levels of *RASSF1C* in the panel of tumor cell lines from lung, colon, glioblastoma, breast, osteosarcoma, and cervical cancers, compared to those detected for *RASSF1A*. This is consistent with previous reports, where the association between the expression levels of the *RASSF1A* and *RASSF1C* isoforms is inversely proportional in lung, breast, and thyroid cancer [36, 37]. Interestingly, the expression levels of *RASSF1A* and *RASSF1‐*AS1 were negatively associated. In this context, Beckedorff et al. determined that *RASSF1‐AS1* may contribute to the *RASSF1A* transcriptional repression by acting as a scaffold RNA, recruiting the PRC2 complex to the promoter region and increasing repressive histone marks such as H3K27me3 [42].

In this study we detected *RASSF1A* promoter hypermethylation in both tumor tissues and liquid biopsies from patients with lung cancer. These results are consistent with those previously reported in studies where the *RASSF1A* methylation marker has demonstrated sensitivity and specificity levels of up to 67% [27, 46, 47, 48, 49]. Concordant with the cell‐line data, *RASSF1A* promoter hypermethylation inversely correlates with expression in lung cancer tissues, supporting its role as an epigenetic mechanism of tumor‐suppressor silencing. Moreover, DNA released into the circulation, via active secretion in extracellular vesicles or through cell death, enables detection of these epigenetic alterations in cell‐free DNA, supporting their potential application in minimally invasive clinical assays [14, 48]. Although the clinical cohort analyzed in this study is limited, the methylation patterns observed are consistent with those detected in larger TCGA lung cancer cohorts, supporting the biological relevance of this alteration. Nevertheless, future studies in larger and clinically balanced cohorts will be required to further evaluate the potential of *RASSF1A* methylation as a biomarker in lung cancer.

## 5 Conclusion

Our study suggests that the *RASSF1* locus undergoes transcription‐specific methylation reprogramming during lung carcinogenesis. *RASSF1A* shows a strong inverse relationship between promoter hypermethylation and gene expression, consistent with its established role as an epigenetically silenced tumor suppressor. In contrast, regulatory regions associated with *RASSF1C* and *RASSF1‐AS1* showed lower levels of DNA methylation accompanied by higher transcriptional expression, with a significant correlation observed for *RASSF1‐AS1*. Specific CpG analyses, together with predictions of transcription factor binding sites, suggest that other regulatory mechanisms may contribute to the differential epigenetic regulation of transcripts within the *RASSF1* locus. Future studies in larger, clinically balanced cohorts will be needed to further evaluate the potential of *RASSF1A* methylation as a biomarker in lung cancer, together with functional approaches to clarify the mechanistic relationships between DNA methylation patterns and the isoform‐specific regulation of *RASSF1* transcripts. Overall, these findings highlight the importance of considering the broader epigenetic architecture of the *RASSF1* locus when evaluating its role in tumor biology and suggest that *RASSF1A* methylation may represent a promising candidate for minimally invasive detection approaches in lung cancer.

## Author Contributions

Adriana Rojas, Teresa Morales‐Ruíz, and Teresa Roldán‐Arjona contributed to conceptualization, data curation, funding acquisition, investigation, methodology, project administration, resources, supervision, and writing – review and editing. Litzy Gisella Bermudez contributed to visualization, formal analysis, methodology, data curation, validation, and writing – original draft. Camila Bernal Forigua, Carmen María Ayala‐Roldán, and Jesús Manuel Romero contributed to data curation, methodology, and writing – review and editing. Rafael Rodríguez contributed to methodology, supervision, and writing – review and editing. Alejandra Cañas contributed to investigation and clinical data curation.

## Funding

This work was funded by Sistema General de Regalías de Colombia (BPIN 2020000100363); Pontificia Universidad Javeriana (20638); and the support of the Enrique Aguilar Benítez de Lugo research plan of the University of Córdoba—UCOIMPULSA 2022 modality.

## Ethics Statement

The study was approved by the Ethics Committee of the Faculty of Medicine at Pontificia Universidad Javeriana (FM‐CIE‐0821‐24) and the Ethics Committee of the Reina Sofia University Hospital (26/07/2012). All procedures were conducted following written informed consent obtained from each participant.

## Consent

The authors have nothing to report.

## Conflicts of Interest

The authors declare no conflicts of interest.

## Supporting information


**Figure S1:** Genomic distribution of CpG sites analyzed by pyrosequencing within the *RASSF1* locus. Schematic representation of the CpG sites included in the pyrosequencing assays within the promoter regions of *RASSF1A*, *RASSF1C*, and *RASSF1‐AS1*, relative to the transcription start site (TSS +1) of each transcript. *RASSF1A* and *RASSF1C* are transcribed from the antisense strand, whereas *RASSF1‐AS1* is transcribed from the sense strand within the *RASSF1* locus. Positions are shown relative to the TSS of each respective transcript.

**Figure S2:** DNA methylation levels at CpG sites within the *RASSF1A* promoter in lung cancer patients from TCGA datasets. (A) DNA methylation levels (*β*‐values) of nine CpG sites detected using the Illumina HumanMethylation450 array located within the *RASSF1A* promoter region in tumor tissue biopsies from patients with lung adenocarcinoma (LUAD). (B) DNA methylation levels (*β*‐values) of nine CpG sites detected using the Illumina HumanMethylation450 array located within the *RASSF1A* promoter region in tumor tissue biopsies from patients with lung squamous cell carcinoma (LUSC). Statistical differences between tumor and normal samples were assessed using the Wilcoxon rank‐sum test, and corresponding *p*‐values are indicated in each panel. All plots were generated using the SMART App platform.

**Figure S3:** DNA methylation levels at CpG sites within the *RASSF1A* promoter across tumor stages in lung cancer patients from TCGA datasets. (A) DNA methylation levels (*β*‐values) of nine CpG sites detected using the Illumina HumanMethylation450 array located within the *RASSF1A* promoter region in tumor tissue biopsies from patients with lung adenocarcinoma (LUAD) stratified by pathological stage (I–IV). (B) DNA methylation levels (*β*‐values) of nine CpG sites detected using the Illumina HumanMethylation450 array located within the *RASSF1A* promoter region in tumor tissue biopsies from patients with lung squamous cell carcinoma (LUSC) stratified by pathological stage (I–IV). Differences across stages were evaluated using one‐way ANOVA, and corresponding *p*‐values are indicated in each panel. All plots were generated using the SMART App platform.

**Table S1:** Oligonucleotides used for quantitative PCR (qPCR), quantitative methylation‐specific PCR (qMSP), and pyrosequencing.

**Table S2:** Transcription factor predicted by MEME analysis in pyrosequenced regions of RASSF1A, RASSF1C and RASSF1‐AS1 with overlap at CpG sites.

## Data Availability

The data that support the findings of this study are available from the corresponding author upon reasonable request.
