# Supplementary figures and images for: Transcript‐Specific DNA Methylation Alterations of the RASSF1 Locus in Cancer Cells
Source: Genes Chromosomes Cancer. 2026 Apr 20;65(4):e70125. doi: 10.1002/gcc.70125 (PMC13125737; doi:10.1002/gcc.70125)

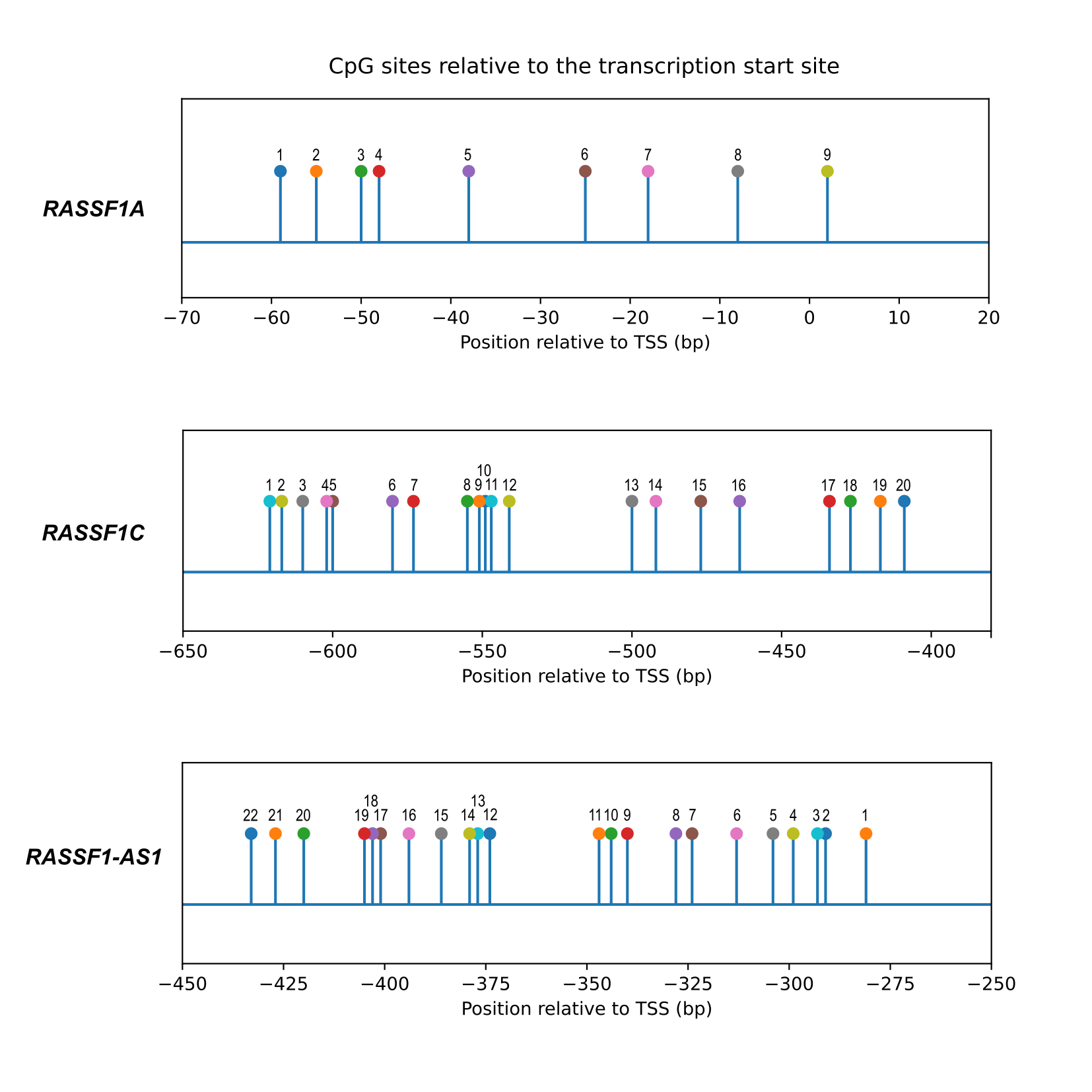

Supplement: Supplementary file 1 — Figure S1: Genomic distribution of CpG sites analyzed by pyrosequencing within the RASSF1 locus. Schematic representation of the CpG sites included in the pyrosequencing assays within the promoter regions of RASSF1A, RASSF1C, and RASSF1‐AS1, relative to the transcription start site (TSS +1) of each transcript. RASSF1A and RASSF1C are transcribed from the antisense strand, whereas RASSF1‐AS1 is transcribed from the sense strand within the RASSF1 locus. Positions are shown relative to the TSS of each respective transcript. [file GCC-65-e70125-s002.png]

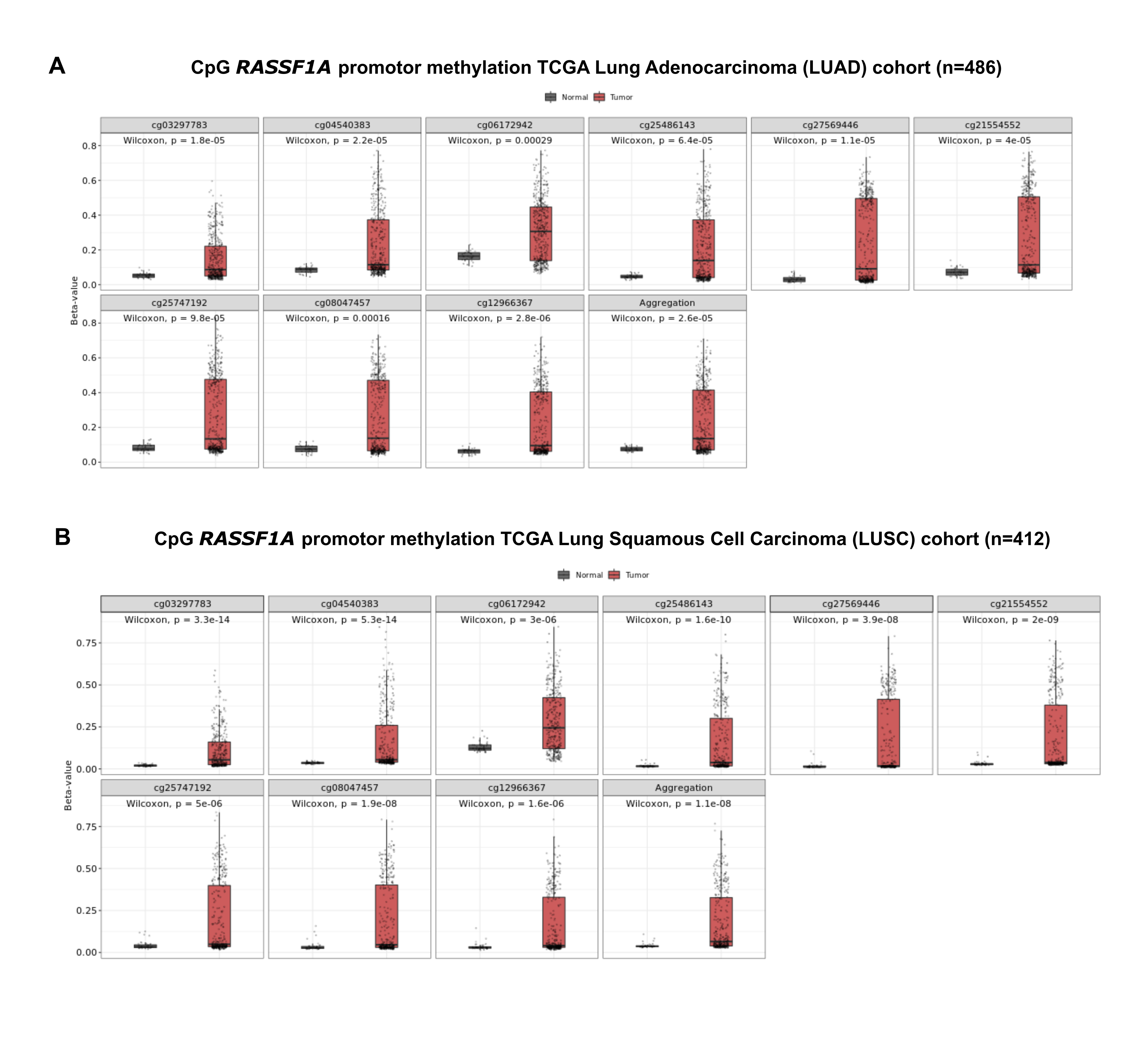

Supplement: Supplementary file 2 — Figure S2: DNA methylation levels at CpG sites within the RASSF1A promoter in lung cancer patients from TCGA datasets. (A) DNA methylation levels (β‐values) of nine CpG sites detected using the Illumina HumanMethylation450 array located within the RASSF1A promoter region in tumor tissue biopsies from patients with lung adenocarcinoma (LUAD). (B) DNA methylation levels (β‐values) of nine CpG sites detected using the Illumina HumanMethylation450 array located within the RASSF1A promoter region in tumor tissue biopsies from patients with lung squamous cell carcinoma (LUSC). Statistical differences between tumor and normal samples were assessed using the Wilcoxon rank‐sum test, and corresponding p‐values are indicated in each panel. All plots were generated using the SMART App platform. [file GCC-65-e70125-s003.png]

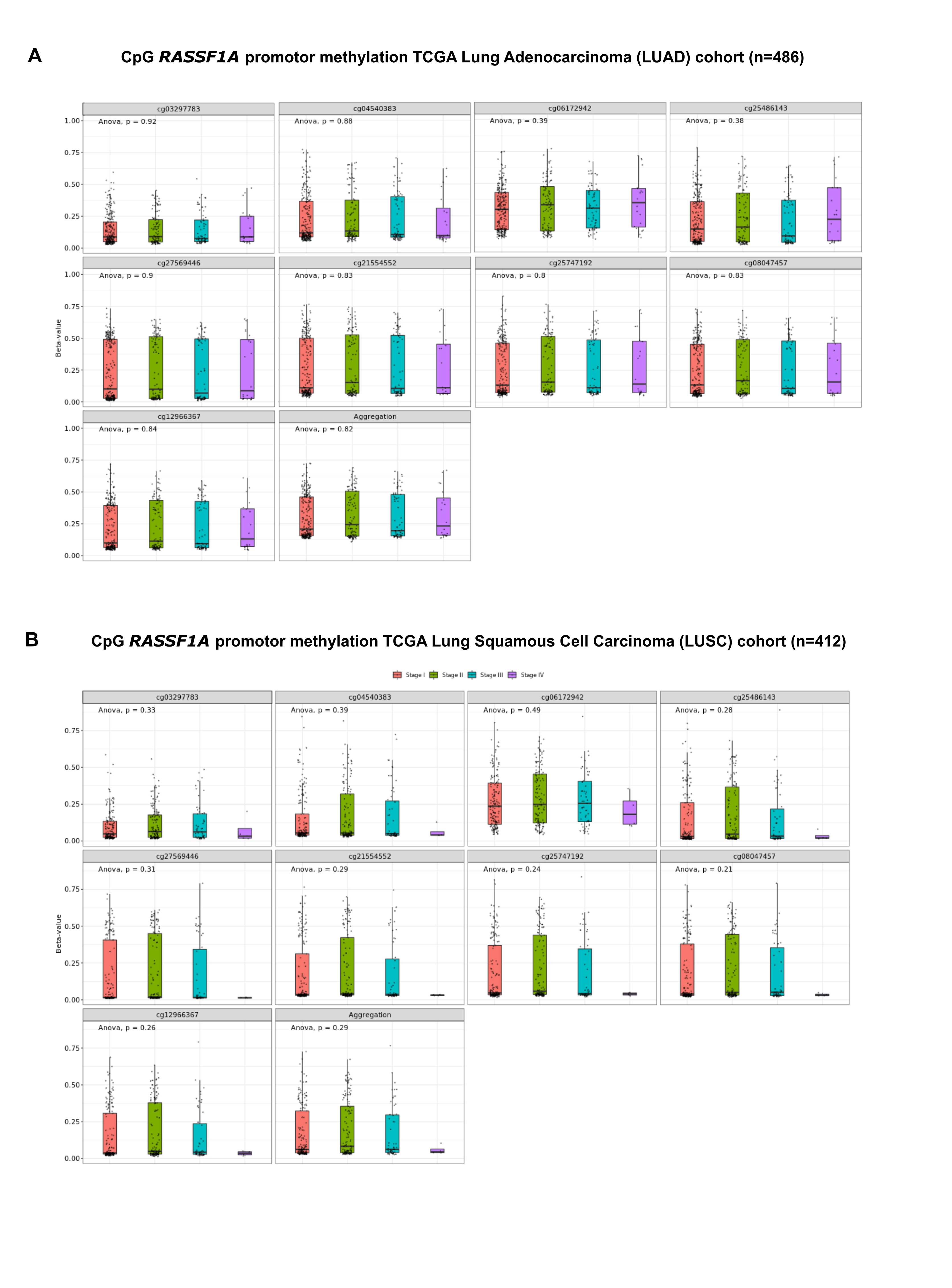

Supplement: Supplementary file 3 — Figure S3: DNA methylation levels at CpG sites within the RASSF1A promoter across tumor stages in lung cancer patients from TCGA datasets. (A) DNA methylation levels (β‐values) of nine CpG sites detected using the Illumina HumanMethylation450 array located within the RASSF1A promoter region in tumor tissue biopsies from patients with lung adenocarcinoma (LUAD) stratified by pathological stage (I–IV). (B) DNA methylation levels (β‐values) of nine CpG sites detected using the Illumina HumanMethylation450 array located within the RASSF1A promoter region in tumor tissue biopsies from patients with lung squamous cell carcinoma (LUSC) stratified by pathological stage (I–IV). Differences across stages were evaluated using one‐way ANOVA, and corresponding p‐values are indicated in each panel. All plots were generated using the SMART App platform. [file GCC-65-e70125-s004.png]
