# Supplementary material for: Transcript‐Specific DNA Methylation Alterations of the RASSF1 Locus in Cancer Cells
Source: Genes Chromosomes Cancer. 2026 Apr 20;65(4):e70125. doi: 10.1002/gcc.70125 (PMC13125737; doi:10.1002/gcc.70125)
Supplement: Supplementary file 4 — Table S1: Oligonucleotides used for quantitative PCR (qPCR), quantitative methylation‐specific PCR (qMSP), and pyrosequencing. [file GCC-65-e70125-s005.docx]

**Table S1:** Oligonucleotides used for quantitative PCR (qPCR), quantitative methylation-specific PCR (qMSP), and pyrosequencing.

|  | Oligonucleotide (5´- 3´) | TM °C | Amplicon length |
| --- | --- | --- | --- |
| qPCR | | | |
| *RASSF1A* | F-ACCTCTGTGGCGACTTCATC | 62 | 158pb |
|  | R-CCAGATGAAGTCGCCACAGA |  |  |
| *RASSF1C* | F-GGAGGCGCCTTCTTTCGAAA | 58 | 278pb |
|  | R-AACCTTGATGAAGCCTGTG |  |  |
| *RASSF1-AS1* | F- CACACTGCTACGCGGACTCTAA | 62 | 110pb |
|  | R- CTGTGCTAGGCGATAGAGATCCA |  |  |
| *GAPDH* | F- AGAAGGCTGGGGCTCATTTG | 60 | 258pb |
|  | R- AGGGGCCATCCACAGTCTTC |  |  |
| *U6* | F-CTCGCTTCGGCAGCACA | 60 | 94pb |
|  | R-AACGCTTCACGAATTTGCGT |  |  |
| 18s | F- ACGGACCAGAGCGAAAGCAT | 60 | 310pb |
|  | R- GCGGGTCATGGGATAACG |  |  |
| Pyrosequencing | | | |
| *RASSF1A* | F-AGTTTGGATTTTGGGGGAGG | 58 | 135pb |
|  | R-CAACTCAATAAACTCAAACTCCCC |  |  |
|  | S-GGGTTAGTTTTGTGGTTT |  |  |
| *RASSF1C* | F- GTAGTGTGAGGTAATTTATTGAGATAG | 56 |  |
|  | R- ACTATAACCTACCCATCCTC |  | 301pb |
|  | S-GAGGTAATTTATTGAGATAGG |  |  |
| *RASSF1-AS1* | F-GAGTAGTGGTTATTGTAGTTAAGAGG |  | 299pb |
|  | R-AACCCCACACCCAACATCTA | 58 |  |
|  | S-GGTTATTGTAGTTAAGAGGAT |  |  |
|  | **qMSP** |  |  |
| *RASSF1A* | F-GGGTTTTGCGAGAGCGCG | 62 | 168pb |
|  | R- GCTAACAAACGCGAACCG |  |  |
| *β-ACTIN* | F-TGGTGATGGAGGAGGTTTAGTAAGT | 58 | 133pb |
|  | R- AACCAATAAAACCTACTCCTCCCTTAA |  |  |
