# Supplementary material for: Transcript‐Specific DNA Methylation Alterations of the RASSF1 Locus in Cancer Cells
Source: Genes Chromosomes Cancer. 2026 Apr 20;65(4):e70125. doi: 10.1002/gcc.70125 (PMC13125737; doi:10.1002/gcc.70125)
Supplement: Supplementary file 5 — Table S2: Transcription factor predicted by MEME analysis in pyrosequenced regions of RASSF1A, RASSF1C and RASSF1‐AS1 with overlap at CpG sites. [file GCC-65-e70125-s001.docx]

**Table S2: Transcription factors predicted by MEME analysis in pyrosequenced regions of RASSF1A, RASSF1C, and RASSF1-AS1 with overlap at CpG sites**

| **Gene** | **Promotor region analyzed** | **Total TF identified** | **Transcriptions factors binding motifs containing CpG sites** | **Matched sequence** | **CpG analyzed** | **p Value** |
| --- | --- | --- | --- | --- | --- | --- |
| ***RASSF1A*** | 50.340.777-50.340.911 | 5 | ZBED4 | CCCGGCCCGC | 2,3 | 0.0000083 |
|  |  |  | KLF15 | GCCCGCGC | 3 | 0.0000264 |
|  |  |  | ZNF213 | GCGCGGGCCGGG | 2,3,4 | 0.0000496 |
|  |  |  | ZNF449 | GGGCCCAACC | 8 | 0.0000791 |
|  |  |  | THRA | CTCCCCCGACATGGCCCG | 8,9 | 0.0000993 |
| ***RASSF1C*** | 50.337.852-50.338.152 | 39 | EBF1 | GCCCCAGGGAG | 3 | 0.00002 |
|  |  |  | TFAP2A | CGCCCTCGGCC | 4 | 0.00002 |
|  |  |  | ZNF770 | CCGCCTCA | 5 | 0.00005 |
|  |  |  | ZBTB24 | CCCAGGTCCC | 6 | 0.00000 |
|  |  |  | ZNF93 | GCGCGCGGCAGCGG | 6 | 0.00000 |
|  |  |  | PLAGL2 | GGGCCCCG | 6 | 0.00010 |
|  |  |  | TFAP2B | GGCCGAGGGCG | 6 | 0.00008 |
|  |  |  | TCFL5 | CCGCGCGC | 6 | 0.00006 |
|  |  |  | FEZF2 | CCCAGCCC | 12 | 0.00009 |
|  |  |  | KLF15 | CCCCGCGC | 13 | 0.00004 |
|  |  |  | ZNF816 | TGGGAACCCGCGGTG | 13 | 0.00010 |
|  |  |  | ZNF682 | AGGGAAGCCCC | 14 | 0.00004 |
|  |  |  | ZNF610 | GTCCCGCTCC | 15 | 0.00004 |
|  |  |  | CTCF | CTGGAGCTCCTCCGCCTCACCGCCAGGCCTCTC | 15 | 0.00008 |
|  |  |  | ZNF669 | GGGCCGCTGCTCGCC | 15 | 0.00003 |
|  |  |  | ZNF331 | TGCGGAGCCT | 16 | 0.00003 |
|  |  |  | ZFP57 | TGCCGCG | 16 | 0.00008 |
|  |  |  | TFAP2E | GCCCCAGGG | 16 | 0.00003 |
|  |  |  | ZKSCAN5 | GGCGGTGAG | 16 | 0.00005 |
|  |  |  | ZBTB24 | CCCGGGGCCC | 17 | 0.00005 |
|  |  |  | ZNF320 | CGCGGGGCCGAGGGCGTATG | 17 | 0.00001 |
|  |  |  | ZNF343 | CCGCTCCACCGCGGGT | 18 | 0.00003 |
|  |  |  | ZNF454 | GGGCCCCGGGACCTGGG | 18 | 0.00004 |
|  |  |  | ZNF460 | TCCTCCGCCTCACCGC | 18 | 0.00004 |
|  |  |  | PAX2 | GCGGTGGAGCGGGACA | 18 | 0.00000 |
|  |  |  | SNAI3 | GCAGGTGAA | 18 | 0.00007 |
|  |  |  | KLF12 | GAGGCGGAG | 18 | 0.00006 |
|  |  |  | ZNF93 | GCGAGCAGCGGCCC | 18 | 0.00009 |
|  |  |  | ZNF135 | CCTCCGCCTCACCG | 18 | 0.00009 |
|  |  |  | ZNF93 | CGCGGCAGCGGTAG | 18 | 0.00005 |
|  |  |  | TFAP2B | GCCCCAGGG | 19 | 0.00006 |
|  |  |  | NFKB1 | AAGGGAAGCCCCA | 19 | 0.00006 |
|  |  |  | ZBED4 | TCGGCCCCGC | 19 | 0.00003 |
|  |  |  | ZNF320 | TGCGGGCCCCGGGACCTGGG | 19 | 0.00001 |
|  |  |  | ZNF93 | GGCAGCGGTAGTGG | 20 | 0.00002 |
|  |  |  | PLAG1 | GAGGGCGTATGGGA | 20 | 0.00001 |
|  |  |  | TFAP2C | GGCCGAGGGCG | 20 | 0.00007 |
|  |  |  | ZNF610 | GGGCCGCTGC | 20 | 0.00005 |
|  |  |  | ZNF454 | GGCCCCGGGACCTGGGC | 20 | 0.00001 |
| ***RASSF1-AS1*** | 50.336.971- 50.337.269 | 28 | ZNF135 | GCTCGAGCTCCGAG | 3 | 0.00001 |
|  |  |  | ZNF460 | CCCCTGGCTCCCACC | 4 | 0.00008 |
|  |  |  | PATZ1 | CCCGGCGCGGC | 5 | 0.00009 |
|  |  |  | ZIC5 | TCCCGCCCGCCGGCA | 6 | 0.00004 |
|  |  |  | TFAP2A | CGCCCGCCGGC | 6 | 0.00002 |
|  |  |  | TFAP2B | GCCCGCCGGCA | 6 | 0.00003 |
|  |  |  | TFAP2C | GCCCGCCGGCA | 6 | 0.00003 |
|  |  |  | E2F8 | CTCCCGCCC | 7 | 0.00008 |
|  |  |  | KLF15 | TCCCGCCC | 7 | 0.00002 |
|  |  |  | KLF10 | AGGGCGGAG | 9 | 0.00007 |
|  |  |  | KLF1 | GGGCGGAG | 9 | 0.00006 |
|  |  |  | PRDM9 | ACGGACCGGGGAGGGCGGAG | 9 | 0.00005 |
|  |  |  | SP2 | AGGGCGGAG | 9 | 0.00005 |
|  |  |  | KLF12 | AGGGCGGAG | 9 | 0.00004 |
|  |  |  | KLF14 | AGGGCGGAG | 9 | 0.000098 |
|  |  |  | KLF7 | GGGCGGAG | 9 | 0.000031 |
|  |  |  | SP4 | AGGGCGGAG | 9 | 0.00002 |
|  |  |  | ZNF343 | CCGCTTCCCCTCCCGC | 10 | 0.000033 |
|  |  |  | SPIB | CCGCTTCCCCTCC | 10 | 0.000049 |
|  |  |  | KLF17 | CCCTCCCGCCCGCC | 10 | 0.000029 |
|  |  |  | MAZ | CCCCTCCC | 10 | 0.000008 |
|  |  |  | KLF4 | CCCCTCCC | 10 | 0.000043 |
|  |  |  | ZNF148 | CCCCTCCCGC | 10 | 0.000001 |
|  |  |  | ZNF530 | GACCGGGGAGGGCG | 11 | 0.000076 |
|  |  |  | ZNF93 | GGACGCGGCAACGG | 13 | 0.000035 |
|  |  |  | ZNF213 | ACCCAGGACGCG | 21 | 0.000072 |
|  |  |  | ZBTB24 | CCCAGGACGC | 21 | 0.000008 |
|  |  |  | PAX2 | CCACCGACCCAGGACG | 22 | 0.000065 |
